# Response to the editorial titled “Public health strategy vs. golden standard for ocular cancer care in Brazil” by Neto, RB

**DOI:** 10.5935/0004-2749.20200104

**Published:** 2024-02-11

**Authors:** Rodrigo Jorge, Priscilla Ballalai Bordon, Enzo Augusto Medeiros Fulco, Daniel Ribeiro, Jefferson Ribeiro, Fernando Chahud, Rafaela Q. Caixeta Faraj, Christian M. C. Campos, Simone Ribeiro A. Almeida, Lucciano Norat, Maristella Bergamo F. dos Reis, Virginia Torres, Zelia M. Correa

**Affiliations:** 1 Ocular Oncology and Vitreoretinal Surgery, Universidade de São Paulo. Riberião Preto, SP, Brazil; 2 Ocular Oncology, Private Practice, São Paulo, SP, Brazil; 3 Private Practice, Manaus, AM, Brazil; 4 Ocular Oncology and Vitreoretinal Surgery, Universidade Estadual do Amazonas, Manaus, AM, Brazil; 5 Ophthalmic Pathologist, Universidade de São Paulo, Ribeirão Preto, SP, Brazil; 6 Ocular Oncology and Vitreoretinal Surgery, Private Practice, Belo Horizonte, MG, Brazil; 7 Ocular Oncology and Medical Retina, Centro Municipal de Oftalmologia, Belo Horizonte, MG, Brazil; 8 Ocular Oncology, Faculdade de Medicina de Marília, Marília, SP, Brazil; 9 Ocular Oncology and Vitreoretinal Surgery, Private Practice, Belém, PA, Brazil; 10 Pediatric Oncologist, Universidade de São Paulo, Ribeirão Preto, SP, Brazil; 11 Ocular Oncology, Private Practice, Recife, PE, Brazil; 12 Ocular Oncology and Vitreoretinal Surgery, Johns Hopkins Medicine, Baltimore, USA

Dear Editor,

We read with great interest a recent editorial titled “Public health strategy vs. golden standard for ocular cancer care in Brazil”^(1)^.

We agree that patient safety needs to be every physician’s primary goal. However, we would like to point out that many ophthalmologists (especially retina specialists) are seeking additional training in ocular oncology and have started multiple centers throughout Brazil in order to serve this unmet need.

In our opinion, there are seven points to be made about this editorial. First, concentrating oncology referrals at a single center in a country with the size and population of Brazil (210 million) is both unreasonable and unsustainable. For this reason, we would recommend that an initiative such as the “OncoPhone” should grow to a country-wide project in telemedicine similar to that established for glaucoma by the Philadelphia initiative^(2)^. This would potentially drive the federally qualified health centers to incorporate this practice in our country, because telemedicine^(3)^ was recently regulated in Brazil, mostly after the onset of the coronavirus disease 2019 pandemic.

Second, the use of WhatsApp and other texting services to share identifiable patient information should be done with caution because of the strict rules regarding patient confidentiality and electronic medical records storage, and because an acceptable level of security cannot be accomplished via WhatsApp chatting. Depending on a country’s federal regulations, using such a system can have potential legal implications.

Third, there are official and efficient ways in Brazil to refer patients to highly specialized physicians. As an example, in the State of São Paulo, there is a system called the CROSS (Central de Regulação de Ofertas de Serviços de Saúde) network that is very effective and eliminates the need for a single institutional phone, thus decentralizing health care. Also, having a well-established and consistently supported referral network allows multiple health institutions, including oncology centers, to work together and in partnership with the state health agency, optimizing and fast-tracking the ref errals. This type of system would avoid several undesirable outcomes from competition between centers that should be working together, such as advertisement of services at the expense others’ reputations and dissemination of anecdotal cases of poor outcomes that most, if not all of us, have unfortunately had to face from time-to-time as physicians^(4)^.

Fourth, we agree that patients deserve access to health care that is timely and efficient. This access is not limited to modern equipment but also includes access to competent professionals whose skills are continually improved after their initial training by the exchange of medical knowledge. Regarding this matter, the Brazilian Council of Ophthalmology, subspecialty societies, and academic centers can join forces to offer continuing medical education, courses, meetings, guidelines for patient referral, and protocols to guide physicians across the country to make uniform management decisions and also stimulate a culture of safe practices^(5)^. This is not an attempt to cover up any physician’s error or unethical conduct, especially if it occurs systematically and unequivocally. Instead, this is an attempt to create uniform procedures that can be agreed upon and serve as a guide for treating patients who cannot travel to the larger centers.

Fifth, transpupillary thermotherapy (TTT) is not the recommended treatment for choroidal melanoma, but it is still a valuable alternative for small melanocytic choroidal tumors, especially those with subretinal fluid and located in the juxta-foveal and paramacular regions where avoidance of radiotherapy and its side effects are significant considerations. TTT is also used to treat other tumors and to supplement plaque brachytherapy in choroidal melanomas. So, in our opinion, to exclude TTT from our therapeutic apparatus is a faulty generalization according to current guidelines^(6,7)^.

Sixth, fine needle aspiration biopsy is a valuable tool in the diagnosis and prognostic evaluation of choroidal tumors that has a very low rate of complications, and it is frequently used even as a confirmatory tool to reassure patients and family members that treatment is necessary^(8)^. We agree that any intraocular biopsy should only be performed by those with adequate training and expertise as the scope of such procedures goes beyond the surgical event.

Seventh, differential diagnosis in ocular oncology is indeed challenging, for example distinguishing advanced or infiltrative retinoblastomas from other conditions such as Coats disease, uveitis, and other causes of vitreous hemorrhage, particularly in older children. We agree that vitrectomy and all intraocular procedures should be avoided in these cases until the possibility of an underlying retinoblastoma is excluded^(9)^. Examination under anesthesia associating ultrasound and magnetic resonance detected 100% of calcifications as opposed to computed tomography that detected 96%^(10)^. Nonetheless, these situations pose diagnostic challenges to many clinicians and again, education and guidelines are the most effective way to minimize that.

Finally, the advancement of all ophthalmology needs collective support, education, and unity. It is useless, and even easy, to criticize our colleagues and point out medical mistakes^(1)^. Instead, we should focus our efforts on solutions that will be mutually beneficial and not concentrated in a single region or center. Currently, several centers throughout the country provide competent, multidisciplinary ocular oncology management for patients and training for the next generation of ophthalmologists specialized in the field of ocular oncology. In this context, Brazilian ophthalmologists expect the ABO journal (Arquivos Brasileiros de Oftalmologia) to be among the main pillars of our profession by providing information that is unbiased, evidence-based rather than personal in nature, peer-reviewed, clinically applicable, and scientifically sound.

## References

[1] Belfort Neto R (2020). Public health strategy vs. golden standard for ocular cancer care
in Brazil. Arq Bras Oftalmol.

[2] Hark LA, Katz LJ, Myers JS, Waisbourd M, Johnson D, Pizzi LT (2017). Philadelphia telemedicine glaucoma detection and follow-up study:
methods and screening results. Am J Ophthalmol.

[3] Brasil, Ministério da Saúde (2020). Dispõe, em caráter excepcional e temporário, sobre
as ações de Telemedicina, com o objetivo de regulamentar e
operacionalizar as medidas de enfrentamento da emergência de
saúde pública de importância internacional previstas no
art. 3º da Lei nº 13.979, de 6 de fevereiro de 2020, decorrente da epidemia
de COVID-19.

[4] Rathi S, Tsui E, Mehta N, Zahid S, Schuman JS. (2017). The current state of teleophthalmology in the United
States. Ophthalmology.

[5] Custer PL, Fitzgerald ME, Herman DC, Lee PP, Cowan CL, Cantor LB (2016). Building a culture of safety in ophthalmology. Ophthalmology.

[6] Singh AD, Kivelä T, Seregard S, Robertson D, Bena JF. (2008). Primary transpupillary thermotherapy of “small” choroidal
melanoma: is it safe?. Br J Ophthalmol.

[7] Shields JA, Shields CL. (2015). Management of posterior uveal melanoma: past, present, and
future: the 2014 Charles L. Schepens lecture. Ophthalmology.

[8] Correa ZM, Faraj RC, Jorge R. (2019). Clinical implications of biopsy for posterior uveal
melanoma. Arq Bras Oftalmol.

[9] Shields CL, Honavar S, Shields JA, Demirci H, Meadows AT. (2000). Vitrectomy in eyes with unsuspected
retinoblastoma. Ophthalmology.

[10] Galluzzi P, Hadjistilianou T, Cerase A, De Francesco S, Toti P, Venturi C. (2009). Is CT still useful in the study protocol of
retinoblastoma?. AJNR Am J Neuroradiol.

